# Lessons Learned From the Pandemic: A Retrospective Analysis of the Impact of COVID-19 in the Routine of a Reference Center for Spine Surgery

**DOI:** 10.7759/cureus.85032

**Published:** 2025-05-29

**Authors:** Yousef Kamel, Galil Osman, Fidaa Al-Shakfa, Leonardo Olijnyk, Jesse Shen, Hao Zhang, Danielle Boulé, Zhi Wang

**Affiliations:** 1 Orthopedic Surgery, Centre Hospitalier de l'Université de Montréal (CHUM), Montreal, CAN

**Keywords:** covid-19, elective spine surgery, orthopedic surgery, surgical case volume, trend analysis

## Abstract

Introduction: The COVID-19 pandemic significantly impacted the operating room schedules and reduced spine-related surgical activity around the world. With the widespread adoption of vaccination and the gradual easing of restrictions in Canada, the healthcare system has largely returned to its pre-pandemic operational capacity.

Methods: This retrospective study evaluated spine surgery activities at Centre Hospitalier de l’Université de Montréal (CHUM), a tertiary academic hospital in Canada. Three continuous but distinct periods were analyzed: pre-pandemic (P1) extended from March 2018 to February 2020; pandemic period (P2), from March 2020 to June 2021; and post-vaccination period (P3), from July 2021 to September 2022. Data on surgical volume, type of surgery (scheduled vs. emergency), mean preoperative waiting time, complication rates, and 30-day mortality rates were compared across the three periods.

Results: A total of 1,775 surgeries were performed during the study period. The monthly surgical volume averaged 34.6 in P1 (820 in total), decreased to 33.8 in P2 (540 in total), and rebounded to 37.7 in P3 (415 in total). Emergency surgeries accounted for 31% of cases in P1, increased to 52% in P2, and decreased to 35% in P3 (p = 0.03) (256, 281, and 144, respectively). Complication rates declined from 10.1% in P1 to 5.6% in P2 and then rose to 10.6% in P3 (p < 0.01) (83, 30, and 44, respectively). The mean preoperative waiting times were 131.3 days in P1, 118.4 days in P2, and 178.8 days in P3 (p < 0.01). The 30-day mortality rate remained less than 1% across all periods.

Conclusion: This work showed the steady volume of surgical activity during the pandemic in a tertiary center. This phenomenon happened largely due to the change in profile in surgical cases. Non-elective cases became more frequent, and elective patients were sparse, probably demonstrating the necessity to deal with cases that otherwise would be treated in community hospitals. Despite this fact, the complication rate decreased during the pandemic, and the mortality rate stayed stable. As the application of the vaccine in the Canadian population, elective spine surgery activity levels were successfully restored.

## Introduction

The COVID-19 pandemic had its fourth anniversary since the World Health Organization (WHO) declared the global outbreak on March 11, 2020, when shutdowns to contain the virus spread around the world severely impacted the health system [1,2]. Despite measures to manage the intense demand of patients with acute respiratory insufficiency, hospitals became easily overwhelmed and elective services were put on hold to mitigate the shock. After consolidation of this scenario, healthcare centers faced a major challenge in balancing resources between providing regular assistance to the population and the concomitant high number of patients diagnosed with coronavirus. In Canada, the initial surge in COVID-19 cases placed unprecedented pressure on the public health system, exposing limitations in capacity and preparedness. As the pandemic evolved, the development and distribution of vaccines became a turning point, playing a crucial role in reducing severe cases and hospitalizations, and enabling gradual resumption of routine medical and surgical services.

The Canadian public healthcare system provides care for the entire population, and central hospitals saw surgical specialties deeply affected by the necessity to redirect human and material resources to patients infected by COVID. Then, it was observed a transition period in which elective operations were gradually rescheduled. This movement was guided by the introduction of institutional safety protocols, global guidelines defined after the first studies on the virus behavior, and rate of COVID bed occupancy at the hospital [3]. Nevertheless, despite significant efforts to increase the efficiency in surgical activity, delays were inevitable. Reasons for that included the required hospital triages, strict cancellation policies, patients’ fear of attending centers emerging from quarantine rules, and the scarcity of healthcare professionals. The latter was, at least partially, related to the burnout of workers often seen at that moment [1,2].

Although spinal trauma cases and metastatic operative diseases seemed to be less affected by those restrictions, the post-pandemic literature showed that the number of degenerative and non-oncologic spine surgeries were considerably reduced following the first COVID wave [3,4]. Some high-volume centers for spinal surgery have registered falls up to 75% from the normal capacity [5-8]. Although this marked decline has been documented by some studies, there is still limited data evaluating the progression of the activity level of spinal surgeries in the period following the lifting of restrictions [8-12].

The objectives of this study are to evaluate the impact of the COVID-19 pandemic and the vaccination rollout on spine surgery activities, including surgical volume, case type, waiting times, and patient outcomes. Specifically, we aim to assess the surgical activity in the spine surgery department at our institution between March 2018 and July 2022, quantify the changes in operative volume, and analyze modifications in the patient profile. In addition, we investigate the effect of the vaccine and the safety practices implemented after the coronavirus outbreak.

## Materials and methods

A retrospective review was performed on all spine surgical cases conducted at Centre Hospitalier de l’Université de Montréal (CHUM), a tertiary academic hospital in Canada, between March 2018 and May 2022. To align with the study’s objectives, the time frame was divided into three distinct periods to assess the impact of the COVID-19 pandemic and subsequent vaccination rollout on surgical activity and outcomes: the pre-pandemic period (March 2018 to February 2020, P1) served as a baseline for evaluating routine surgical activity and patient characteristics; the pandemic period (March 2020 to June 2021, P2) captured the effects of the outbreak and the introduction of public health restrictions; and the post-vaccination period (July 2021 to May 2022, P3) allowed for analysis of changes following widespread vaccination, including potential recovery of surgical volume and normalization of case profiles. These time frames were defined based on key public health milestones, specifically the onset of pandemic-related restrictions in March 2020 and the initiation of second-dose COVID-19 vaccinations for adults in Canada in June 2021. This temporal framework enabled targeted analysis of how surgical volume, case mix, waiting times, and patient outcomes evolved across each stage of the pandemic response.

The data analyzed in each period included the number of spine surgeries performed, the priority of surgery (elective or non-elective), presence of postoperative complications, mortality in 30 days following procedure, and length of stay after surgery. Intraoperative parameters such as blood loss, operating time, and number of instrumented levels were also assessed.

The study included all patients who underwent spinal surgery during the defined study period performed by one of five spine fellowship-trained surgeons at our institution (two neurosurgeons and three orthopedists). Data were obtained from the hospital’s electronic medical records system and surgical scheduling database, which comprehensively document all operative activity at the institution. Only index coded spinal surgeries were considered. More particular operations performed by one of the surgeons were analyzed case by case to define inclusion. Exclusion criteria were procedures with primary pathology unrelated to the spine, cases with incomplete operative data, and reoperations during the same admission to avoid duplication of clinical outcomes.

Data were collected and organized in spreadsheets by medical archivist in our institution and posteriorly reviewed independently by medical trained authors (YK, GO, LO, and MK) before statistical analysis. Incoherent information, imprecise values, and outstanding variables were checked individually to ensure accuracy and solid metrics.

Elective patients were considered to be all those who had been seen by a surgeon during a scheduled consultation and had a procedure programmed with a priority greater than a seven-day delay. Non-elective was any patient scheduled for surgery in seven days after outpatient consultation, evaluated in the emergency room and admitted for surgery, and transferred from another hospital for surgical admission or any patient that was already admitted for any other medical reason and were indicated for spinal surgery at the same admission after assessment by a surgeon. Complications were defined as an intraoperative unexpected event or postoperative surgical or clinical complication within 30 days of the operation.

Statistical analysis was performed using the IBM SPSS Statistics for Windows, Version 27.0 (released 2020, IBM Corp., Armonk, NY). Student’s T-test was used to compare means, and Chi-square test was used to compare proportions. Multivariate analysis was performed to identify predictive factors based on the outcomes of the univariate analysis. The significance level was set at 0.05.

## Results

A total of 1,776 surgeries were performed during the period. During P1, the mean number per month was 34.2. This trend decreased to 33.8 surgeries/month in P2 and rose to 37.7 in P3. There was no significant difference among the periods. Moreover, 31% of the interventions were non-elective surgeries during P1, 52% in period P2, and 35% in P3. These oscillations were statically significant for the two change of terms (p = 0.003). The mean preoperative waiting time for an elective procedure also differ among the periods and was 131.3 days in P1, 118.44 days during P2, and 178.75 days in P3 (p < 0.001). Thirty days mortality averaged less than 1% in all three periods. Data are summarized in Table 1.

**Table 1:** Profile of spine surgeries from March 2018 to September 2022 *Significant change from the previous period of time (i.e., comparing pandemic to pre-pandemic and post-vaccination to pandemic periods) for p < 0.05. + Significant change from pre-pandemic time to post-vaccination time. Statistical analysis was performed using the IBM SPSS Statistics software, Student’s T-test, Chi-square test, and multivariate analysis.

	Pre-pandemic (%)	Pandemic (%)	Post-vaccination (%)
Elective surgeries			
Total	820	540	415
Per month (mean)	34.2	33.8	37.7
Non-elective surgeries	256 (31%)	281 (52%)	144 (35%)
Preoperative waiting time	131.3 days	118.4 days*	178.8 days*+
30-day mortality rate	<1%	<1%	<1%

The complication rate passed from 10.1% in P1 to 5.6% in P2 and then to 10.6% in P3. Those differences were statically significant (p < 0.001). The duration of surgery was 201 minutes in average during P1, decreased not significantly to 190 minutes in P2, and remained stable in 189 minutes during P3. The length of stay had a mean duration of 14.7 days in pre-pandemic, fell significantly to 11.6 during the pandemic, and were shortest after vaccination, with 8.2 days. Instrumented levels for procedure were 2.9, 2.7, and 2.3 for P1, P2, and P3, respectively. The mean blood loss in P1 was 597cc, diminished significantly to 457cc during P2, and was 528cc in post-vaccination time. The operative characteristics are represented in Table 2.

**Table 2:** Intraoperative surgical variables and length of stay *Significant change from previous period of time (i.e., comparing pandemic to pre-pandemic and post-vaccination to pandemic periods) for p < 0.05. **The mean number of vertebrae that were operated on during spinal surgery in each period. + Significant change from pre-pandemic time to post-vaccination time. Statistical analysis was performed using the IBM SPSS Statistics software, Student’s T-test, Chi-square test, and multivariate analysis.

	Pre-pandemic (%)	Pandemic (%)	Post-vaccination (%)
Operating time (minutes)	201.9	190.9	189.6
Length of stay (days)	14.6	11.6^*^	8.2^*+^
Levels instrumented**	2.9	2.7	2.3
Blood loss (cc)	597	457^*^	528
Complication rate	83 (10.1%)	30 (5.6%)^* ^	44 (10.6%)^*^

## Discussion

The effects of the COVID-19 pandemic on the healthcare sector continues to be studied across all nations. The pandemic has had a significant impact on surgical volume and most hospitals experienced drastic reduction in their elective operative activity. For spine surgery, it was not different. Our study aimed to quantify and describe the effect of the pandemic on the number of elective spinal surgeries performed in a tertiary center by comparing regular activity in the period just before the pandemic, throughout the pandemic, and during the period when restrictions could be slowly lifted after the vaccine became available.

The outbreak of the pandemic has led to a considerable decrease in elective procedures worldwide due to the necessity of redistributing human, material, and financial resources. We analyzed three different but continuous periods at our institution to better understand how COVID-19 impacted the routine of a non-trauma spine reference center. Data from the 24 months preceding the pandemic was collected to serve as a reference and compare with a period subsequent to the first outbreak observed in March 2020. A second period was then determined by the absence of active immunization in the population. During this phase, virus spread could only be controlled by physical and behavior measures in hospital. The third interval was counted from the start of the second-dose vaccination in Canada, when the population became increasingly immunized against COVID. Although surgical activity during the pandemic was not defined solely by the vaccination status of the population and was instead determined by terms updated weekly by hospitals and the government, we chose this point as a transition to study the impact in the health system and its behavior in the scenario where only physical barriers and educational protocol could defend individuals against the infection.

**Figure 1:**
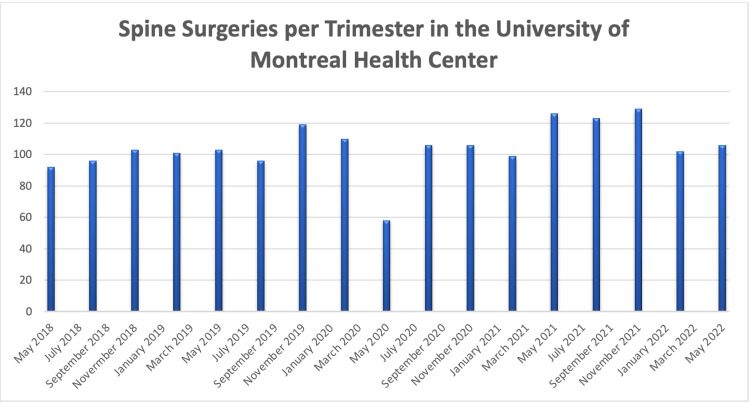
Despite the impact of COVID-19 in the profile of spine operations, the volume of surgeries was affected only for a brief period of time. In the total number of procedures, there was a rebound less than two months after the state of pandemic established in Canada and it has remained stable over the following months. This image was created by the authors.

 As expected, following the surge of cases during March 2020 in Canada, there was a decrease in the number of elective and total spinal surgeries at the first moment. However, when we compared the pre-pandemic period with the numbers of the first 15 months of pandemic, there was no significant decline in total volume of operations (Figure 1). On the other hand, we observed a significant change in the profile of these operations. Until the pandemic, non-electives surgeries accounted for 31% of the cases. During the peak of the pandemic, emergencies comprised 52% of all procedures. This change in profile has a potential influence on the surgical team routine, the organization of the surgical room and, eventually, the availability of time and human resources for non-urgent patients, normally scheduled on elective basis.

Although the surgical team remained available to meet the demand for spine procedures, the initial weeks of the COVID-19 outbreak required a period of adaptation before the surgical load could be safely accommodated in the operating rooms. The abrupt reduction in spinal surgeries during this time was largely due to the need for implementation of safety protocols to minimize viral transmission, the reorganization of hospital infrastructure, and restrictions on social mobility following the lockdown. Despite the shift in the surgical case profile, the hospital quickly regained its operative capacity in terms of numbers, and within the following trimester, the number of spine surgeries rebounded to pre-pandemic levels. It is reasonable to consider that the surgical capacity offered by the healthcare system was significantly affected during the pre-vaccination period, which likely contributed to the shift in the operative profile observed at our tertiary center. Nevertheless, the rapid reorganization undertaken at our institution highlights its ability to respond effectively to systemic stress and maintain operating room functionality when adequate resources and infrastructure are in place.

The rise in the proportion of emergency spine surgeries can be partly attributed to the closure of community centers for such procedures. While tertiary centers typically handle both routine and more complex elective cases, community and non-academic hospitals tend to focus on patients with less advanced conditions and center their resources on patients with acute presentation, traumas and who can be helped with less cumbersome operations. However, as the pandemic has stressed resources of the entire system, patients who would normally be assisted in peripheral hospitals have had to be redirected to larger centers where there was still capacity to run operative rooms. Therefore, despite the reduction in elective cases, our hospital needed to supply a greater demand for non-elective spinal surgeries from the referral area seen during COVID.

Interestingly, we noticed a significant reduction in the complication rate from 10.1% in the pre-pandemic period to 5.6% during the pandemic. This decline may be partly due to the lower complexity of emergency cases, as opposed to elective procedures, which often involve deformity corrections, advanced degenerative diseases, and higher-risk patients who were not prioritized during that time. This accompanied a decline in intraoperative blood loss and hospital stay, although we observed no difference in the average number of levels instrumented in the spine. Complication rates returned from 5.6% during the peak of the pandemic to 10.6% in the post-vaccination period. One explanation for this is the resumption of the typical list of non-urgent surgeries in P3, characterized by more demanding routine cases, such as the elective list for deformity cases and advanced degenerative pathologies. Furthermore, during the pandemic, safety protocols including pre-operative assessments have become more rigorous, which may have contributed for the reduction in post-op complication rates. Nonetheless, the significant decrease in the length of admission is likely linked to reasons such as the shift in the profile of operative cases, the understandable pressure from the system to make beds available attempting to minimize the patients’ exposure to COVID infection in the hospital environment, and the possibility of repatriation toward a peripheral center as the number of patients transferred for spine surgery became more prevalent. This point may potentially bias our assessment to complications, since some of them can still occur during the admission but we are limited access data when the stay prolongs in another institution. Taken together, although our study does not fully elucidate certain findings, such as the reduction in complications and hospital length of stay, our observations provide a foundation for future investigations by public health authorities and open space for strategic development of high-demand surgical centers.

Safety workflows at our tertiary center were also implemented. The hospital administration worked collaboratively with surgeons to implement a pre-admission triage system to ensure a prioritization of patients in critical conditions. In addition, our facilities are relatively new (inaugurated in 2017), and all the patients have their stay in individual rooms with own bathroom. The advanced air filtration system also played important role in limiting COVID-19 propagation [13].

Post-vaccination days also inherited a longer waiting time for elective procedures. The backlog due to blocked operative rooms resulted in an average delay of 178.8 days for surgeries compared to 131.3 before coronavirus. Other reasons for this trend may involve the preoperative respiratory virus screenings and a variety of other regulations that healthcare facilities have adopted in response to the first COVID-19 outbreak. For example, the reduced number of consultations in the outpatient clinic also contributed to see the preoperative waiting time at its lowest during the pandemic.

Despite the variety of responses seen to the pandemic from governments, COVID-19 has hit the different health systems universally. Decreases in the number of spine elective spine surgeries were systematically reported around the globe following 2020. In some institutions, a third of elective spine surgeries cancelled due to COVID-19 infections were still waiting to be performed in the eight months following the resuming of a regular OR schedule [14]. Abramovic et al. published the results of a questionnaire addressed to spinal surgical professionals, revealing a reduction in elective spinal surgeries due to increased preventive measures implemented during the pandemic, which aimed to allocate additional resources for acutely ill COVID-19 patients [6]. Some preventive measures included a 45% reduction in outpatient work, an increase of telemedical care by 73%, and a reduced availability of medical equipment by 75%, as well as medical staff by 30%.

Numerous studies documented a marked decline of elective spine surgeries in tertiary hospitals [8-12]. In some instances, the level of surgical activity did not return to baseline until the third and fourth quarter of 2020 [15]. While acute spinal trauma cases and metastatic operative diseases seemed to be less affected by those restrictions, cases involving degenerative spine pathologies were often re-scheduled, eventually repeated times, as they were deemed low priority [11]. As a result, the waiting time for operation saw steep raise with time, as we observed in this study for patients in need of spine surgery in the post-vaccination period.

In Canada, hospitals implemented new safety protocols to try to cautiously restore regular daily operations [16], but the occurrence of several waves after the first outbreak prevented a consistent return to normality until the widespread of immunization. The pattern of surgeries observed in our study confirms this. As of July 2021, the national successful campaign of COVID-19 vaccination that reached the target of 50% of fully protected residents [17] led to an alleviation in the restrictions for scheduling elective spinal procedures could be maintained steadily.

Other elements have influenced the spectrum of patients presenting to the hospital during COVID. In Taiwan, Hsu et al. attributed a drop in the number of knee arthroplasty by 60% due to the population fear to attend healthcare facilities crowed by infected patients [4]. Here, in Canada, surveys have confirmed that patients were hesitant to go to tertiary hospitals for surgical care due to the presence of COVID-19-infected patients treated in place [18]. Although this may represent a bias in patient selection during the pre-vaccination period, and could partially explain the decline in waiting times from P1 to P2, our study demonstrates that efforts to sustain the availability of elective procedures during the pandemic were effective. As anticipated, in P3, with social restrictions easing and hospital operations gradually returning to normal, the waiting time for spine surgery experienced a notable increase.

This fear was not only shared by patients. The anxiety levels among healthcare professionals in Canada reached unprecedent levels [1]. The levels of infection among health workers were another point of concern during allocation of human resources during the pandemic. Nicholas et al. found a COVID-19 seroprevalence up to 32.0% by 2021 among healthcare workers in hospitals where outbreaks in the personnel were registered [2]. How this specifically affected the variables measured in this study is unclear but certainly the safety of any staff in the healthcare system was a concern in hospital protocols and progressively improved with the lessons learned during the pandemic.

Limitations

This study includes the inherent limitations of retrospective chart reviews. Although patients and surgical protocols are typically digitalized today, missing data remains a possibility, and retrospective designs are susceptible to selection bias and limit the ability to control for confounding variables. In additioj, the lack of detailed classification of surgical complexity and absence of comorbidity data restricts the depth of analysis, particularly when interpreting outcomes such as complication rates and length of stay. Another limitation concerns the specific profile of any high-volume tertiary center. Our institution serves a defined region in Canada within a virtually universal public healthcare system and is characterized by a broad intake of spinal emergencies, while acute trauma cases are referred to another regional center. Finally, we chose to determine a specific time period for comparison of data as discussed in the study, but the literature is scarce in examining the impact of COVID in framed time windows. Rather than thinking that this could be arbitrary, we believed that a variety of alternatives could also be explored on the topic.

## Conclusions

This retrospective study analyzes the post-pandemic effects of COVID-19 on elective spine surgery from 2020 to 2022. Results suggest a minimal overall surgical activity reduction during the pandemic. Our hospital, being a tertiary center, received an increase in referrals of emergency spine surgeries, which reflected in a shift of operative patient profile. There was also reduction in the complications rates and length of stay. These can be explained by a reduced complexity of emergency surgeries compared to adult spine deformity surgery commonly performed in our center. Strict COVID-19 prevention rules were applied during P2, as witnessed by the stable 30-day mortality rate. As pre-pandemic spine surgery activity levels were successfully restored, efforts should be targeted toward reducing increased waiting time seen in P3 due to the elective surgery rate reduction in P2. Having said that, COVID-19 was a unique event in modern society and a prospective study for a pandemic cannot be elaborated for obvious reasons.
